# Role of lncRNA Xist-miR-124-CCL2 axis in HIV Tat-mediated microglial activation and neuroinflammation

**DOI:** 10.3389/fimmu.2025.1558842

**Published:** 2025-05-08

**Authors:** Palsamy Periyasamy, Seema Singh, Abiola Oladapo, Muthukumar Kannan, Shilpa Buch

**Affiliations:** Deparment of Pharmacology and Experimental Neuroscience, University of Nebraska Medical Center, Omaha, NE, United States

**Keywords:** CCL2, HIV Tat, lncRNA Xist, microglia, miR-124, neuroinflammation

## Abstract

**Introduction:**

HIV proteins, such as the Transactivator of transcription (Tat), mediate neuroinflammation in the central nervous system by promoting the release of pro-inflammatory cytokines and chemokines. Long noncoding RNAs (lncRNAs) regulate gene expression by sponging microRNAs (miRs), but their role in HIV Tat-mediated microglial activation remains poorly understood. This study aimed to investigate the involvement of the lncRNA Xist–miR-124–CCL2 axis in HIV Tat-exposed microglial cells.

**Methods:**

Mouse primary microglial cells were exposed to HIV Tat, and the expression of lncRNA Xist, miR-124, and CCL2 was evaluated using qPCR, Western blotting, and ELISA. Dual-luciferase reporter and Argonaute immunoprecipitation assays were used to confirm molecular interactions. Functional experiments involved lncRNA Xist silencing and miR-124 overexpression. *In vivo* validation was performed using doxycycline-inducible HIV Tat transgenic mice.

**Results:**

HIV Tat significantly upregulated lncRNA Xist and downregulated miR-124 expression in mouse primary microglial cells. miR-124 was identified as a direct target of lncRNA Xist and the 3′-UTR of CCL2. Silencing lncRNA Xist or overexpressing miR-124 reduced HIV Tat-induced CCL2 expression and microglial activation. *In vivo* studies corroborated these findings, with doxycycline-fed iTat mice showing elevated lncRNA Xist and CCL2 levels and reduced miR-124 expression in the frontal cortex.

**Discussion:**

Our findings identify a novel regulatory axis whereby HIV Tat-induced upregulation of lncRNA Xist sponges miR-124, leading to CCL2 overexpression and microglial activation. Targeting the lncRNA Xist–miR-124–CCL2 pathway may represent a promising therapeutic strategy to mitigate neuroinflammation associated with NeuroHIV.

## 1 Introduction

HIV infection remains a chronic condition despite the remarkable success of combination antiretroviral therapy (cART) in reducing peripheral viral replication and significantly extending the lifespan of people living with HIV. Paradoxically, however, the limited penetrance of cART in the brain allows the central nervous system (CNS) to harbor early viral proteins and serve as a sanctuary for latent reservoirs, thereby contributing to neuroinflammation. Microglia, the resident immune cells of the CNS, are key targets of HIV, serving both as virus reservoirs while also being a source of neurotoxic viral proteins, such as HIV Transactivator of transcription (Tat). HIV Tat has been well-recognized to mediate the activation of inflammatory pathways – a central feature of HIV-associated neurocognitive disorders, also referred to as NeuroHIV. NeuroHIV, that afflicts almost 30-60% of people living with HIV (PLWH), is characterized as a spectrum of cognitive impairments ranging from mild motor deficits to overt dementia in its severe form. Despite advances in treatment, the underlying mechanisms driving chronic neuroinflammation and NeuroHIV remain less well-understood, limiting the development of effective therapeutic interventions.

Microglia play a dual role in the CNS, maintaining homeostasis under physiological conditions but also becoming activated in response to infection or injury. This activation is tightly regulated to prevent excessive inflammation in the brain, however, under prolonged exposure to toxic stimuli, such as those encountered in NeuroHIV, the balance shifts to overt neuroinflammation, leading to excessive release of proinflammatory cytokines and chemokine such as CCL2 (also known as MCP-1; monocyte chemoattractant protein-1). CCL2 is a key player in sustaining the inflammatory environment in the brain, by recruiting peripheral immune cells across the blood-brain barrier (BBB), and hence perpetuating neuroinflammation. Elevated levels of CCL2 in the CNS cells such as astrocytes, microglia, and neurons have been associated with the progression of NeuroHIV (1–4). While there are reports on HIV Tat-mediated induction of MCP-1 (5, 6), the precise molecular mechanisms regulating its expression in microglia remains poorly defined and presents a critical gap in our understanding of the disease pathogenesis.

MicroRNAs (miRs), which are small non-coding RNAs, serve as key regulators of gene expression by targeting messenger RNAs (mRNAs) for degradation or translation repression. They are integral to numerous cellular processes, including inflammation, apoptosis, and oxidative stress, which are central to neurodegenerative disorders such as NeuroHIV (7–10). Recent literature has identified several miRs that are differentially expressed and contribute to NeuroHIV by promoting neuroinflammatory responses (11–13) as well as the neurotoxic effects, leading to neuronal and synaptic damage (14, 15). MiR-124 is a highly expressed miR in the CNS and plays a pivotal role in maintaining microglial homeostasis (16–18). By targeting proinflammatory signaling pathways, such as NF-κB and STAT3, miR-124 prevents the transition of microglia from a resting state to an activated phenotype (8, 19). Dysregulation of miR-124 has been linked to increased microglial activation and upregulation of inflammatory mediators, including CCL2 (20–24). Although the role of miR-124 in maintaining microglial quiescence is well-documented, the factors contributing to its dysregulation in NeuroHIV remain poorly understood.

Recent research highlights the role of long non-coding RNAs (lncRNAs) as upstream regulators of miRs, with significant implications in various cellular processes such as neuroinflammation and cellular proliferation. LncRNAs are non-coding RNA molecules that regulate gene expression through various mechanisms, including serving as competitive endogenous RNAs (ceRNAs) or as “miR sponges”. By binding to specific miRs, lncRNAs sequester these small molecules, thereby preventing their interaction with target mRNAs. The ceRNA mechanism enables lncRNAs to modulate activity of various key miRs and, in turn, influence downstream gene expression. In the context of neuroinflammatory diseases, several lncRNAs have been identified as key modulators of inflammation. For example, lncRNA Cox2, regulated by nuclear factor κB signaling, plays a pivotal role in microglial immune function (25). Studies have shown that extracellular vesicles derived from morphine-exposed astrocytes can transfer lncRNA Cox2 to microglia, leading to Toll-like receptor 7 activation, upregulation of lncRNA Cox2, and subsequent impairment of microglial phagocytic activity. Intriguingly, these effects were reversible through intranasal delivery of extracellular vesicles containing lncRNA Cox2 siRNA, underscoring the therapeutic potential of targeting lncRNA pathways in neurodegeneration (25). Another example is MALAT1 that has been shown to regulate neuroinflammation by interacting with miR-125b (26). While many other lncRNAs have been implicated in the pathogenesis of Alzheimer’s disease and cerebral infarction (27, 28), the role of lncRNAs in NeuroHIV, particularly their influence on microglial activation and inflammatory pathways, remains underexplored.

Given that LncRNA Xist has been implicated in various neuroinflammatory conditions, it is plausible that it may contribute to the pathogenesis of NeuroHIV by modulating miR-124 activity, a microRNA known to play a critical role in maintaining microglial quiescence and regulating neuroinflammatory pathways (18). However, its specific involvement in NeuroHIV remains unexplored, highlighting the need for further investigation. Previous studies have shown that lncRNA Xist can act as a ceRNA, sponging miRs and altering their availability for gene regulation (29–32). In Alzheimer’s disease, lncRNA Xist promotes neuroinflammation by suppressing pathways involved in amyloid-beta clearance (28), while in the ischemic brain injury, it has been linked to microglial polarization and inflammatory responses (27). These findings suggest that lncRNA Xist could likely have broader implications for neuroinflammation across diverse neurological conditions. The dysregulation of the lncRNA Xist-miR-124 axis in microglia could represent a novel mechanism underlying chronic neuroinflammation in NeuroHIV. By elucidating this pathway, we aim to provide new insights into the molecular drivers of microglial activation in PLWH. This research is particularly significant given the central role of microglial activation in the pathogenesis of NeuroHIV and the potential to target these mechanisms therapeutically. This study aims to investigate the role of lncRNA Xist in regulating miR-124 and CCL2 expression during HIV Tat-mediated microglial activation. Specifically, we aim to determine whether lncRNA Xist acts as a ceRNA to modulate miR-124 activity, thereby influencing inflammatory signaling and microglial activation. By addressing these questions, this study has the potential to uncover novel targets that could mitigate neuroinflammation and cognitive decline in PLWH, thus contributing to improved outcomes in NeuroHIV.

## 2 Materials and methods

### 2.1 Isolation of mouse primary microglia

Primary microglia were isolated from the brains of 1–3-day-old C57BL/6 mouse pups using a standard mixed glial culture protocol (8, 33, 34). Briefly, brain tissues were dissected, mechanically dissociated, digested with trypsin, and the resulting cell suspension was filtered through a 40 µm mesh to remove debris. The cells were then centrifuged and seeded in cell culture flasks containing Dulbecco’s modified Eagle’s medium (DMEM) supplemented with 10% fetal bovine serum (FBS), 1X penicillin/streptomycin 0.25 ng/ml mouse macrophage colony stimulating factor (mCSF, Cat No. PHC9504, Thermo Fisher Scientific, USA) and OPI media supplement (Cat No. O5003, Millipore-Sigma, Burlington, MA, USA). After 10–14 days, microglia were separated from the mixed glial cultures by mild agitation. The purity of the microglia was confirmed through immunostaining with the Iba1 antibody, achieving a purity of over 95%.

### 2.2 TaqMan miR assay

Total RNA, including small RNA, was extracted from control and treated microglia using the Quick-RNA™ MiniPrep Plus kit (Cat. No. R1058, Zymo Research, Orange, CA, USA) following the manufacturer’s instructions. The quantification of miR-124 expression was performed using TaqMan MicroRNA Assays (miR-124 Assay ID: 001182; U6 snRNA Assay ID: 4427975; Thermo Fisher Scientific, Waltham, MA, USA). RNA samples were reverse transcribed using the TaqMan MicroRNA Reverse Transcription Kit, and qPCR was conducted using the TaqMan Universal PCR Master Mix on a real-time PCR system. Expression levels were normalized to U6 small nuclear RNA, which served as an endogenous control.

### 2.3 Quantitative polymerase chain reaction

Total RNA was isolated from both control and treated microglia using the Quick-RNA™ MiniPrep Plus kit (Cat. No. R1058, Zymo Research, Orange, CA, USA), following the protocol provided by the manufacturer. Complementary DNA (cDNA) was synthesized using the iScript™ Reverse Transcription Supermix for RT-qPCR (Cat. No. 1708841, Bio-Rad, Hercules, CA, USA), in accordance with the manufacturer’s guidelines. The expression levels of lncRNA Xist (Assay ID. Mm01232884_m1) and CCL2 (Assay ID. Mm00441242_m1) were assessed using TaqMan Gene Expression Assays. qPCR was performed with the TaqMan^®^ Universal PCR Master Mix, no AmpErase^®^ UNG (Cat. No. 4324018, Thermo Fisher Scientific, Waltham, MA, USA) on an Applied Biosystems^®^ QuantStudio™ 3 Real-Time PCR System (Applied Biosystems, Grand Island, NY, USA). Each reaction was conducted in triplicate, with six independent experiments performed. *Gapdh* (Assay ID: Mm99999915_g1; ThermoFisher Scientific, Waltham, MA, USA) served as the housekeeping control for normalization, and the relative fold change in gene expression was calculated using the 2^−ΔΔCT^ method.

### 2.4 Western blotting

Protein lysates were prepared from the control and treated microglia using RIPA buffer supplemented with protease (Cat. No. 78429, Thermo Fisher Scientific, Waltham, MA, USA), and phosphatase inhibitors (Cat. No. 78426, Thermo Fisher Scientific, Waltham, MA, USA). Protein concentrations were quantified using the BCA assay (Cat. No. 23227, Thermo Fisher Scientific, Waltham, MA, USA), and equal amounts of proteins were loaded onto 10% SDS-PAGE gels. After electrophoresis, proteins were transferred onto PVDF membranes. Membranes were blocked in 5% non-fat milk and incubated overnight at 4°C with primary antibodies against CD11b (Cat. No. NB110-89474, Novus Biological Company, Centennial CO, USA; 1:5000 dilution), CCL2 (Cat. No. ab25124, Abcam Boston, MA, USA; 1:1000 dilution) and β-actin (Cat. No. sc-47778 HRP Santa Cruz Biotechnology, Dallas, TX, USA; 1:5000 dilution). After washing, membranes were incubated with HRP-conjugated secondary antibodies and visualized using an enhanced chemiluminescence detection system. Band intensities were quantified using ImageJ software (35), normalized to β-actin.

### 2.5 miR profiler PCR array

To analyze differential miRNA expression, the miScript™ miRNA PCR Array (Mouse Neurological Development and Disease, Cat. No. 331221 MIMM-107ZA, Qiagen, Hilden, Germany) was used in a 96-well plate format. Total RNA was extracted from control and HIV Tat-treated mouse primary microglia using the miRNeasy Mini Kit (Qiagen, Hilden, Germany). RNA was then reverse transcribed into cDNA using the miScript II RT Kit (Qiagen, Hilden, Germany) following the manufacturer’s instructions. qPCR was carried out using the miScript SYBR Green PCR Kit in an Applied Biosystems^®^ QuantStudio™ 3 Real-Time PCR System (Applied Biosystems, Grand Island, NY, USA). Normalization was performed using specific housekeeping miRNAs included in the array. All procedures, including array preparation and data analysis, were conducted as per the manufacturer’s instructions to ensure accurate profiling of miRNA expression in microglia.

### 2.6 miR target validation

Validation of miR-124 interaction with CCL2 was performed using an RNA immunoprecipitation (RIP) assay (miRNA Target IP Kit, Cat. No. 25500, Active motif, Carlsbad, CA, USA). Lysates from miR-124 mimic transfected microglia were prepared and incubated with anti-Ago2 antibodies to pull down the RNA-induced silencing complex (RISC). Co-immunoprecipitated RNAs were purified, and qPCR was performed to determine the fold enrichment, confirming the binding of miR-124 to the target sequence of CCL2 mRNA.

### 2.7 Enzyme-linked immunosorbent assay

The concentration of CCL2 protein secreted into the culture supernatant was quantified using a commercially available ELISA kit (Cat. No. ab20897, Abcam Boston, MA, USA). Samples were processed according to the manufacturer’s protocol, and absorbance was measured at 450 nm using a microplate reader. A standard curve was generated to calculate CCL2 concentrations in the samples.

### 2.8 miR-124 mimic transfection

Mouse primary microglial cells were transfected with miR-124 mimics (Cat. No mirVana™ 4464066 ThermoFisher Scientific, Waltham, MA, USA) using Lipofectamine RNAiMAX (Cat. No. L3000015, Thermo Fisher Scientific, Waltham, MA, USA) following the manufacturer’s protocol. Briefly, cells were plated in 6-well plates and transfected with 50 nM miRNA mimics diluted in Opti-MEM (Cat. No. 31985070, ThermoFisher Scientific, Waltham, MA, USA). Transfection efficiency was confirmed by assessing miR-124 expression levels using qPCR.

### 2.9 lncRNA array

Expression profiling of lncRNAs was conducted using the RT² lncRNA PCR Array (Cat. No. 330721, GeneGlobe ID: LAMM-001Z, Qiagen, Hilden, Germany). Total RNA was isolated from the control and HIV Tat exposed mouse primary microglia and was reverse transcribed, and the array was performed according to the manufacturer’s instructions. Normalization was performed using specific housekeeping genes included in the array. All procedures, including array preparation and data analysis, were conducted as per the manufacturer’s instructions to ensure accurate profiling of lncRNA expression in microglia.

### 2.10 Luciferase reporter assay

The interaction between miR-124 and lncRNA Xist was analyzed using a luciferase reporter assay. Constructs containing the wild-type and mutant sequences of lncRNA Xist binding sites for miR-124 were cloned into a luciferase reporter vector (Cat. No. E1330; Promega, Madison, WI, USA). HEK293 cells were co-transfected with these constructs and miR-124 mimics using Lipofectamine 3000 (Cat. No. L3000015, ThermoFisher Scientific, Waltham, MA, USA). Luciferase activity was measured 48 hours post-transfection using a Dual-Luciferase Reporter Assay System (Cat. No. E2920; Promega, Madison, WI, USA) and normalized to Renilla luciferase activity, as per manufacturers’ instructions.

### 2.11 siRNA transfection for lncRNA Xist

Silencing of lncRNA Xist in the mouse primary microglia was performed using siRNAs targeting lncRNA Xist (Cat No. 4392420, Assay ID. n519032, ThermoFisher Scientific, Waltham, MA, USA). Cells were transfected with 50 nM siRNA using Lipofectamine RNAiMAX in Opti-MEM medium according to the manufacturer’s protocol. Next, the transfected cells were exposed to HIV Tat (50 ng/mL; 24 hours), and total RNA and proteins were extracted for miR-124 expression and microglial activation. Knockdown efficiency was also confirmed by qPCR.

### 2.12 Animals

Doxycycline (Dox)-inducible HIV Tat transgenic (iTat) mice were used in this study. Male mice aged 3–4 months were housed under controlled conditions with regulated temperature and humidity, following a 12-hour light/dark cycle. The animals were provided with unrestricted access to standard food and water. To induce the expression of the Tat protein, the experimental groups were fed a diet enriched with Dox (Cat No. TD.01306, Harlan Teklad, Indianapolis, IN; 625 mg/kg) for 21 days, while control groups received a standard diet. After the induction period, the mice were euthanized, and brain tissue, specifically the cortex, was collected. All procedures involving animals adhered to the guidelines set forth by the Institutional Animal Care and Use Committee at the University of Nebraska Medical Center and the National Institutes of Health.

### 2.13 Statistical analysis

All experiments were conducted in biological replicates (N=6 for *in vitro* and N=3 for *in vivo*), with results expressed as mean ± standard error of the mean (SEM). Statistical analyses were performed using GraphPad Prism (version 10.4.1, San Diego, CA, USA). Comparisons between two groups were made using unpaired Student’s t-tests, while one-way ANOVA was used for comparisons among multiple groups, followed by appropriate *post hoc* tests. A p-value of less than 0.05 was considered statistically significant.

## 3 Results

### 3.1 HIV Tat induced cellular activation and downregulated miR-124 expression in mouse primary microglia

**Figure 1:**
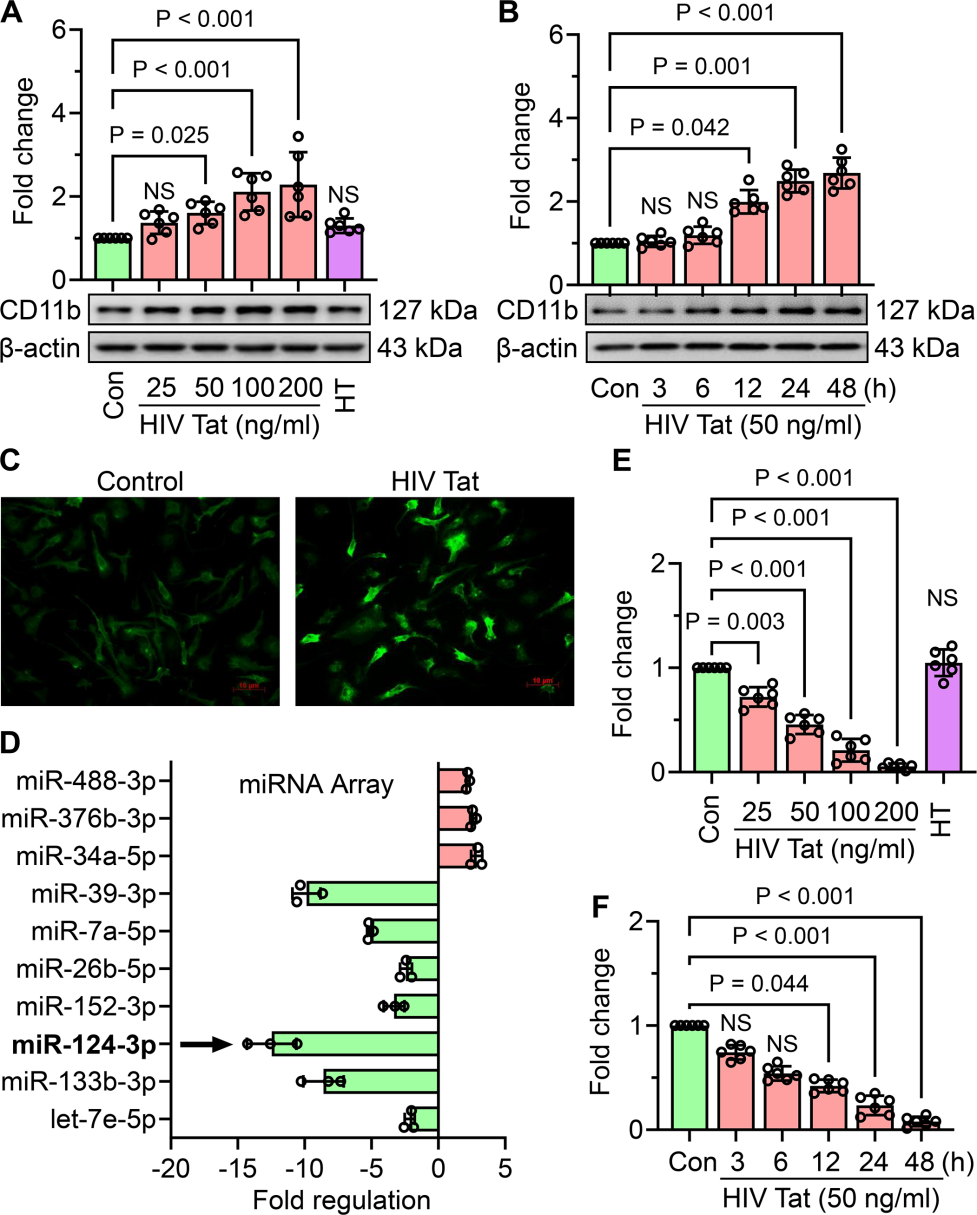
HIV Tat induces cellular activation and suppresses miR-124 expression in mouse primary microglial cells. **(A)** Representative western blot image and corresponding bar graph demonstrate a dose-dependent increase in CD11b expression in mouse primary microglial cells exposed with varying concentrations of HIV Tat for 24 h **(B)** Representative western blot image and bar graph illustrate the time-dependent increase in CD11b expression in mouse primary microglial cells exposed to HIV Tat (50 ng/mL). C) Immunocytochemical staining reveals the expression of CD11b in mouse primary microglial cells exposed with HIV Tat (50 ng/mL). **(D)** Bar graph from the miScript PCR Array showing differential expression of miRs in HIV Tat-exposed mouse primary microglial cells, with a notable downregulation of miR-124. **(E, F)** Bar graphs validate the dose- and time-dependent downregulation of miR-124 expression in HIV Tat-exposed mouse primary microglial cells, as determined by TaqMan qPCR. β-actin served as the internal control across all experiments. Data are presented as mean ± SEM from six independent experiments. Statistical analysis was performed using nonparametric Kruskal–Wallis one-way ANOVA followed by Dunn’s *post hoc* test. NS, no significant difference.

To evaluate the effects of HIV Tat on microglial activation, mouse primary microglial cells were treated with increasing concentrations of HIV Tat (25–200 ng/mL) or heat-inactivated HIV Tat (50 ng/mL) for 24 hours. Western blot analysis of CD11b, a key microglial activation marker, demonstrated a significant dose-dependent increase in CD11b expression, with activation becoming prominent at concentrations of 50 ng/mL and higher (**Figure 1A**). In contrast, heat-inactivated HIV Tat did not induce a similar response, thus confirming the specificity of active HIV Tat in promoting microglial activation. To examine the temporal effects of HIV Tat, mouse primary microglial cells were exposed with 50 ng/mL of HIV Tat for varying durations (0–48 hours). CD11b expression was significantly upregulated in a time-dependent manner, with a marked increase observed starting at 12 hours and sustained through later time points (**Figure 1B**). Based on these findings, 50 ng/mL of HIV Tat for 24 hours was selected as the optimal dose- and time-point for subsequent experiments. Immunocytochemistry further confirmed the activation of microglia, with increased CD11b staining in cells treated with HIV Tat (50 ng/mL, 24 hours) compared to untreated controls (**Figure 1C**). To investigate the molecular mechanism(s) underlying HIV Tat-induced microglial activation, a miScript miR PCR Array for Mouse Neurological Development and Disease was performed. Exposure of mouse primary microglial cells to HIV Tat (50 ng/mL, 24 hours) revealed significant differential expression of several microRNAs, including miR-124, a well-known regulator of microglial quiescence (**Figure 1D**). Notably, miR-124 levels were markedly decreased following HIV Tat exposure. Validation of these findings using TaqMan qPCR demonstrated a dose - (**Figure 1E**) and time-dependent (**Figure 1F**) downregulation of miR-124 expression in mouse primary microglial cells exposed to HIV Tat. These results indicate that HIV Tat induces microglial activation through mechanism(s) involving the suppression of miR-124, thus implicating miR-124 dysregulation as a potential driver of microglial activation in the context of NeuroHIV.

### 3.2 HIV Tat-mediated downregulation of miR-124 targets the 3′-UTR of C-C motif chemokine ligand 2

**Figure 2:**
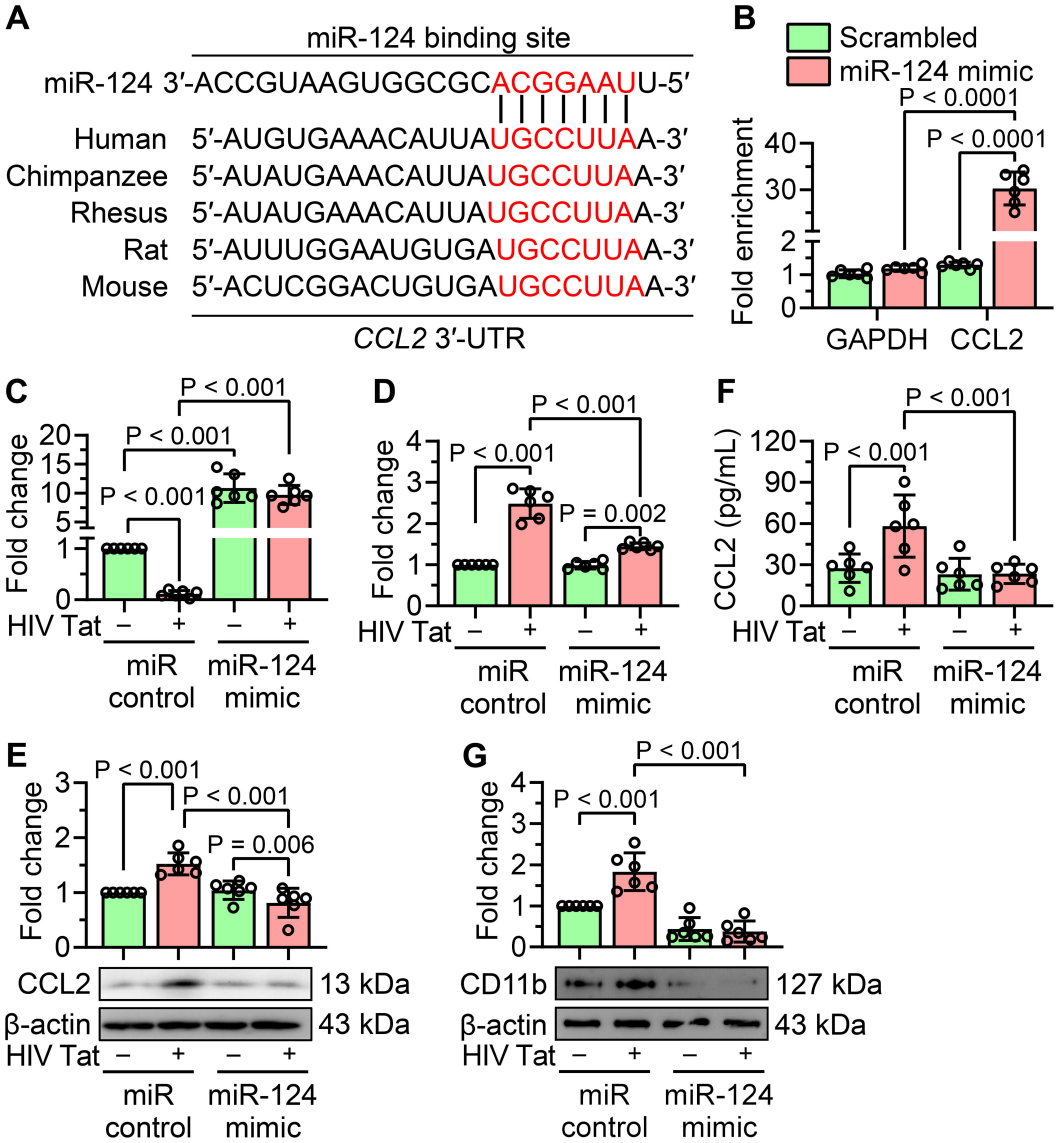
miR-124 attenuates CCL2 expression and HIV Tat-induced microglial activation. **(A)** *In silico* analysis illustrates the conservation of miR-124 binding sites within the 3′-UTR of CCL2 across multiple species. **(B)** miR target validation assay confirms the enrichment of CCL2 mRNA as a target of miR-124 in argonaute immunoprecipitation, compared to total RNA isolated from mouse primary microglial cells transfected with miR-124 mimic or scrambled control. GAPDH was used as a housekeeping control. **(C)** qPCR analysis confirms the transfection efficiency of miR-124 in mouse primary microglial cells transfected with the miR-124 mimic, followed by exposure to HIV Tat (50 ng/mL, 24 hours). **(D–F)** qPCR **(D)**, western blot **(E)**, and ELISA **(F)** analyses demonstrate significant suppression of CCL2 expression in mouse primary microglial cells transfected with the miR-124 mimic after treatment with HIV Tat (50 ng/mL, 24 hours). **(G)** Representative western blot image and bar graph show a significant reduction in CD11b expression in mouse primary microglial cells transfected with miR-124 mimic and subsequently exposed with HIV Tat (50 ng/mL, 24 hours). β-actin was used as the internal control for all experiments. Data are expressed as mean ± SEM from six independent experiments. Statistical significance was assessed using nonparametric Kruskal–Wallis one-way ANOVA followed by Dunn’s *post hoc* test.

miRNAs primarily regulate gene expression by binding to the 3′-untranslated regions (3′-UTRs) of their target mRNAs, suppressing their expression. To identify potential miR-124 targets associated with HIV Tat-induced microglial activation, we utilized the TargetScan database and identified CCL2 as a putative target of miR-124. CCL2 is a well-characterized chemokine implicated in microglial activation associated with NeuroHIV. Based on these findings, we hypothesized that HIV Tat-mediated downregulation of miR-124 leads to upregulation of CCL2, in turn, contributing to inflammation and microglial activation. To support this hypothesis, we first examined the sequence conservation of the miR-124 binding site within the 3′-UTR of CCL2. *In silico* analysis revealed that the miR-124 binding site within the 3′-UTR of CCL2 is highly conserved across various species, strongly supporting CCL2 as a direct target of miR-124 (**Figure 2A**). To experimentally validate this interaction, we performed an Argonaute (Ago) immunoprecipitation assay, a widely used method for miRNA target validation. Mouse primary microglial cells were transiently transfected with a miR-124 mimic or a miR control. Immunoprecipitation of mRNAs bound to the Ago complex was performed using an anti-Ago antibody, and the associated mRNAs were quantified by qPCR. As shown in **Figure 2B**, CCL2 mRNA was significantly enriched in the Ago complex of cells transfected with the miR-124 mimic, thus confirming CCL2 as a direct target of miR-124. However, miR-124 may regulate multiple transcripts depending on the cellular context.

To evaluate the impact of miR-124 on both CCL2 expression and microglial activation, mouse primary microglial cells were transiently transfected with either a miR-124 mimic or a miR control and exposed to HIV Tat (50 ng/mL, 24 hours). Transfection efficiency of the miR-124 mimic was confirmed by qPCR (**Figure 2C**). Quantification of CCL2 mRNA using qPCR and protein levels using western blotting, and ELISA revealed that HIV Tat significantly upregulated CCL2 expression at both the transcript and protein levels in control cells. However, overexpression of miR-124 significantly suppressed CCL2 expression in both untreated and HIV Tat-exposed cells (**Figures 2D–F**). Next, we explored the role of miR-124 in modulating HIV Tat-induced microglial activation. Mouse primary microglial cells were transfected with a miR-124 mimic or a miR control and subsequently exposed to HIV Tat (50 ng/mL, 24 hours). Overexpression of miR-124 significantly attenuated HIV Tat-induced upregulation of microglial activation markers compared to cells transfected with the miR control (**Figure 2G**). These findings collectively demonstrated that miR-124 plays a crucial role in suppressing CCL2 expression and mitigating microglial activation in the context of HIV Tat exposure.

### 3.3 HIV Tat induced the expression of lncRNA Xist in mouse primary microglial cells

**Figure 3:**
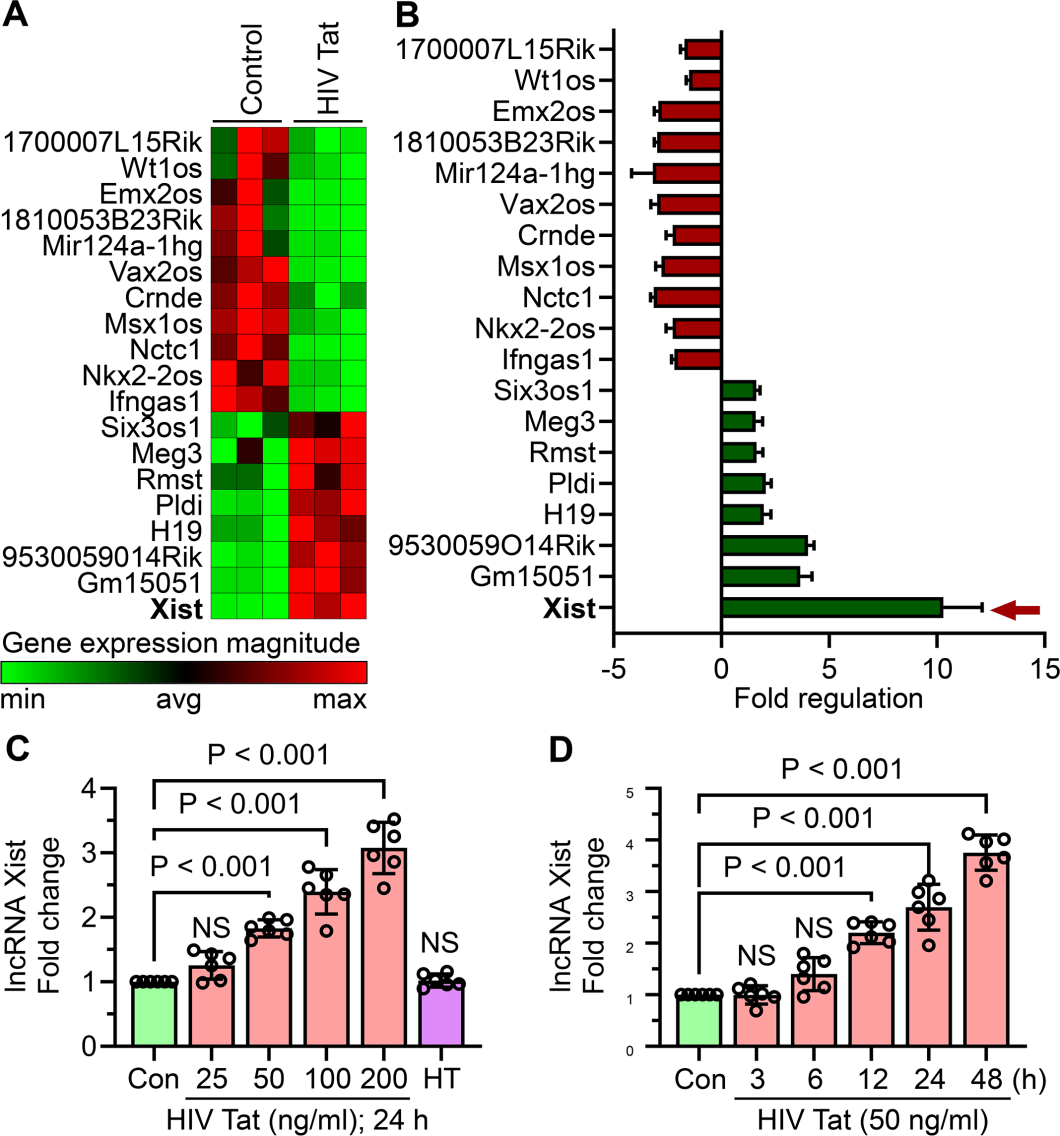
HIV Tat induces lncRNA Xist expression in mouse primary microglial cells. **(A)** RT² lncRNA PCR array profiling reveals differential expression of lncRNAs in mouse primary microglial cells treated with HIV Tat (50 ng/mL) for 24 hours (n=3). **(B)** Bar graph shows the expression levels of various lncRNAs, including lncRNA Xist, in mouse primary microglial cells following HIV Tat exposure (50 ng/mL) for 24 hours (n=3). **(C)** qPCR analysis demonstrates the dose-dependent expression of lncRNA Xist in mouse primary microglial cells treated with varying concentrations of HIV Tat (25–200 ng/mL) for 24 hours. **(D)** qPCR analysis shows the time-dependent expression of lncRNA Xist in mouse primary microglial cells exposed to HIV Tat (50 ng/mL) for different time points. GAPDH served as the housekeeping control. Data are expressed as mean ± SEM from six independent experiments. Statistical significance was assessed using nonparametric Kruskal–Wallis one-way ANOVA followed by Dunn’s *post hoc* test. NS, no significant difference.

To explore the potential involvement of lncRNAs in HIV Tat-induced microglial activation, we used the RT² lncRNA PCR Array Mouse lncFinder (Qiagen) to profile dysregulated lncRNAs in mouse primary microglial cells exposed to HIV Tat (50 ng/mL, 24 hours). Our analysis revealed that lncRNA Xist was significantly upregulated following the exposure of cells to HIV Tat (**Figure 3A**). Additionally, other lncRNAs, including Gm15051 and 9530059O14Rik, were also upregulated (**Figure 3B**). To further evaluate lncRNA Xist expression, we performed dose- and time-dependent studies in mouse primary microglial cells exposed to HIV Tat. Cells exposed to varying doses of HIV Tat (25–200 ng/mL) for 24 hours showed a dose-dependent increase in lncRNA Xist expression, with significant upregulation starting at 50 ng/mL (**Figure 3C**). Importantly, heat-inactivated HIV Tat did not alter lncRNA Xist expression, confirming the role of active HIV Tat in driving these changes (**Figure 3C**). Next, we assessed the time-dependent expression of lncRNA Xist by exposing cells to 50 ng/mL of HIV Tat for different durations (3, 6, 12, 24, and 48 hours). We observed a significant increase in lncRNA Xist expression as early as 6 hours post-treatment, which persisted through 48 hours (**Figure 3D**). These findings demonstrate that HIV Tat induces lncRNA Xist expression in a dose- and time-dependent manner, suggesting its involvement in the epigenetic mechanisms underlying microglial activation and neuroinflammation.

### 3.4 LncRNA Xist directly interacts with miR-124 in mouse primary microglial cells

**Figure 4:**
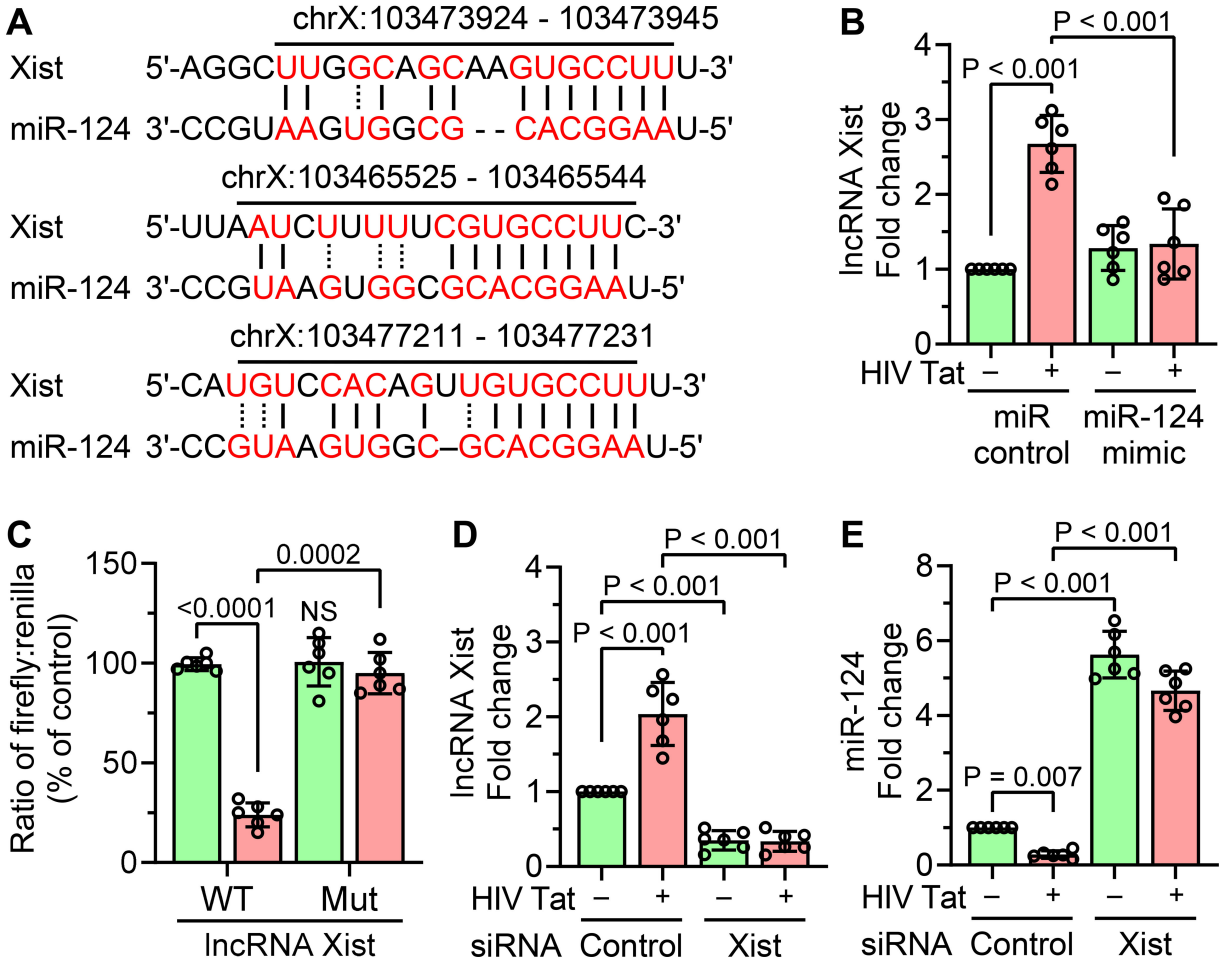
Interaction between lncRNA Xist and miR-124. **(A)** *In silico* analysis using the starBase v2.0 database predicts the binding sites of miR-124 on lncRNA Xist. **(B)** qPCR analysis shows the expression of lncRNA Xist in mouse primary microglial cells overexpressing miR-124 mimic, followed by HIV Tat exposure (50 ng/mL, 24 hours). **(C)** Dual-luciferase reporter assay confirms the direct interaction between lncRNA Xist and miR-124 in mouse primary microglial cells. **(D)** qPCR analysis demonstrates the transfection efficiency of lncRNA Xist siRNA in mouse primary microglial cells transfected with lncRNA Xist siRNA, followed by HIV Tat exposure (50 ng/mL, 24 hours). **(E)** qPCR analysis shows increased expression of miR-124 in mouse primary microglial cells transfected with lncRNA Xist siRNA and exposed with HIV Tat (50 ng/mL, 24 hours). GAPDH served as the housekeeping control. Data are presented as mean ± SEM from six independent experiments. Statistical significance was assessed using nonparametric Kruskal–Wallis one-way ANOVA followed by Dunn’s *post hoc* test. NS, no significant difference.

Using the starBase v2.0 database, we next identified lncRNA Xist as a potential ceRNAs for miR-124, implying its role in the regulation of microglial activation (**Figure 4A**). To investigate this interaction, mouse primary microglial cells were transfected with a miR-124 mimic and subsequently exposed to HIV Tat (50 ng/mL, 24 hours). As shown in **Figure 4B**, miR-124 overexpression significantly reduced HIV Tat-induced upregulation of lncRNA Xist, thus indicating an inverse relationship between lncRNA Xist and miR-124. To further confirm the direct binding of miR-124 to lncRNA Xist, a dual-luciferase reporter assay was conducted. Mouse primary microglial cells were co-transfected with a luciferase plasmid containing the wild-type (WT) miR-124 binding site of lncRNA Xist and a miR-124 mimic. This resulted in a marked decrease in luciferase activity (**Figure 4C**). In contrast, no reduction in luciferase activity was observed when cells were co-transfected with a luciferase plasmid containing a mutated miR-124 binding site on lncRNA Xist, thereby underscoring the specificity of the interaction (**Figure 4C**). We also explored the effect of lncRNA Xist knockdown on miR-124 regulation in HIV Tat-exposed mouse primary microglia. Efficient knockdown of lncRNA Xist using siRNA was confirmed by qPCR (**Figure 4D**). Importantly, silencing lncRNA Xist significantly upregulated miR-124 expression in cells exposed to HIV Tat, whereas scrambled siRNA had no such effect (**Figure 4E**). These findings collectively demonstrated that lncRNA Xist functions as a ceRNAs for miR-124, sequestering miR-124 in the presence of HIV Tat and contributing to microglial activation and inflammation.

### 3.5 Gene silencing of lncRNA Xist prevented HIV Tat-induced CCL2 expression and cellular activation in mouse primary microglial cells

**Figure 5:**
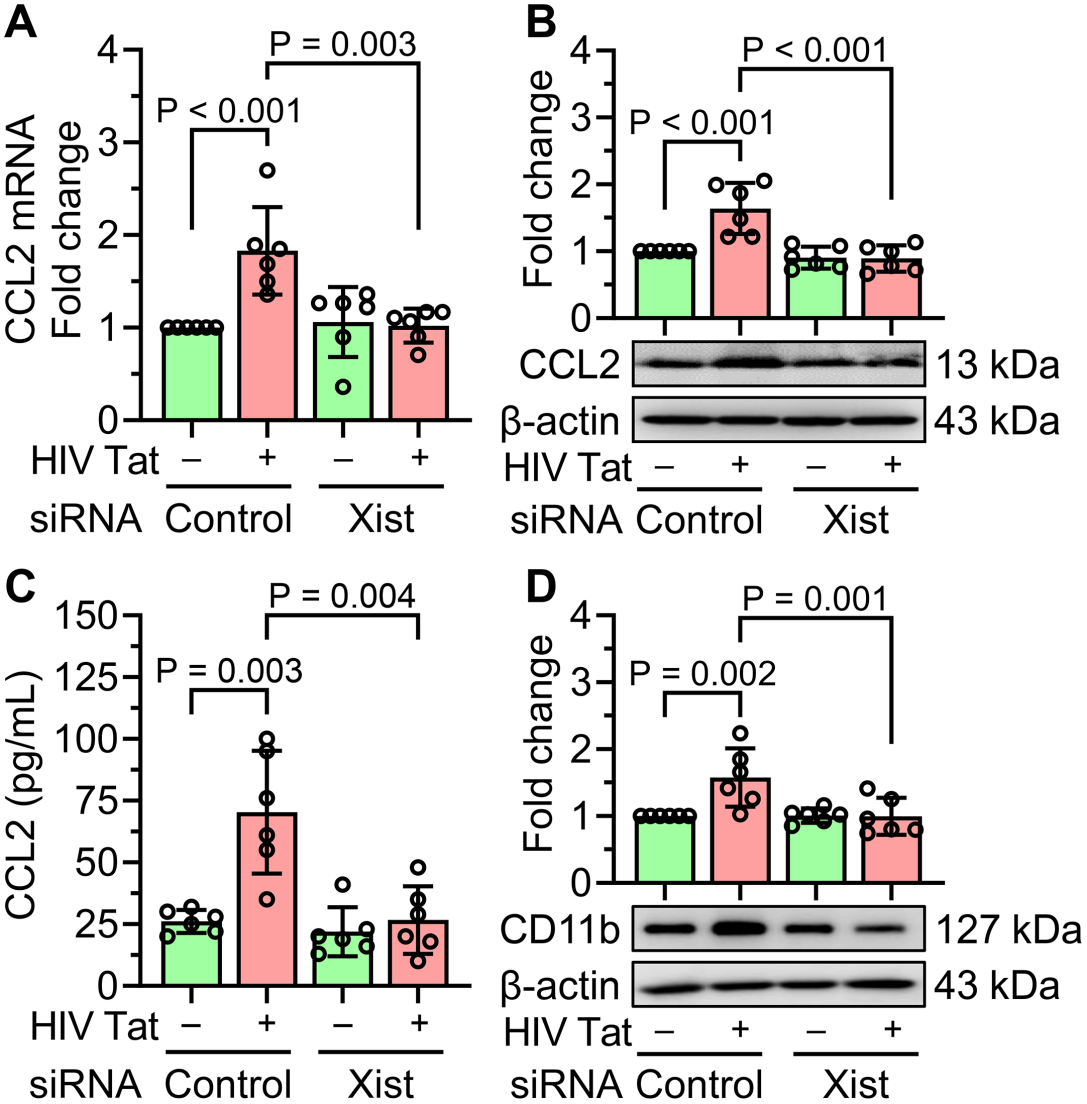
Gene silencing of lncRNA Xist prevents HIV Tat-induced CCL2 expression and microglial activation. **(A)** qPCR analysis shows the mRNA expression of CCL2 in mouse primary microglial cells silenced with lncRNA Xist followed by HIV Tat (50 ng/mL) exposure for 24 hours. **(B)** representative western blot image and bar graph analysis confirm the expression of CCL2 protein in mouse primary microglial cells silenced with lncRNA Xist followed by HIV Tat (50 ng/mL) exposure for 24 hours. **(C)** ELISA quantification confirms the expression of CCL2 protein in mouse primary microglial cells silenced with lncRNA Xist followed by HIV Tat (50 ng/mL) exposure for 24 hours. **(D)** representative western blot and bar graph quantification confirms the expression of CCL2 protein in mouse primary microglial cells silenced with lncRNA Xist followed by HIV Tat (50 ng/mL) exposure for 24 hours. β-actin was used as the internal control for all experiments. Data are presented as mean ± SEM from six independent experiments. Statistical significance was assessed using nonparametric Kruskal–Wallis one-way ANOVA followed by Dunn’s *post hoc* test.

To investigate the regulatory role of lncRNA Xist in HIV Tat-induced upregulation of the chemokine CCL2, we silenced lncRNA Xist in mouse primary microglial cells using siRNA and subsequently exposed the cells to HIV Tat (50 ng/mL, 24 hours). As shown in **Figure 5A**, transfection with lncRNA Xist siRNA, but not scrambled siRNA, significantly downregulated the HIV Tat-induced increase in CCL2 mRNA expression. This reduction in CCL2 expression was also evident at the protein level, as demonstrated by western blotting (**Figure 5B**), and in the amount of CCL2 released into the culture supernatant, as measured by ELISA (**Figure 5C**). In addition to suppressing CCL2 expression, lncRNA Xist silencing also significantly reduced the expression of the microglial activation marker CD11b in HIV Tat-exposed cells (**Figure 5D**). Compared to control cells transfected with scrambled siRNA, lncRNA Xist silencing effectively abrogated HIV Tat-induced microglial activation. These findings thus suggest that lncRNA Xist plays a critical role in mediating HIV Tat-induced CCL2 expression and microglial activation. Gene silencing of lncRNA Xist attenuated these effects, highlighting its role as a potential therapeutic target to mitigate neuroinflammation in the context of NeuroHIV.

### 3.6 Validation of *in vitro* findings in the frontal cortices of doxycycline-fed iTat mice

**Figure 6:**
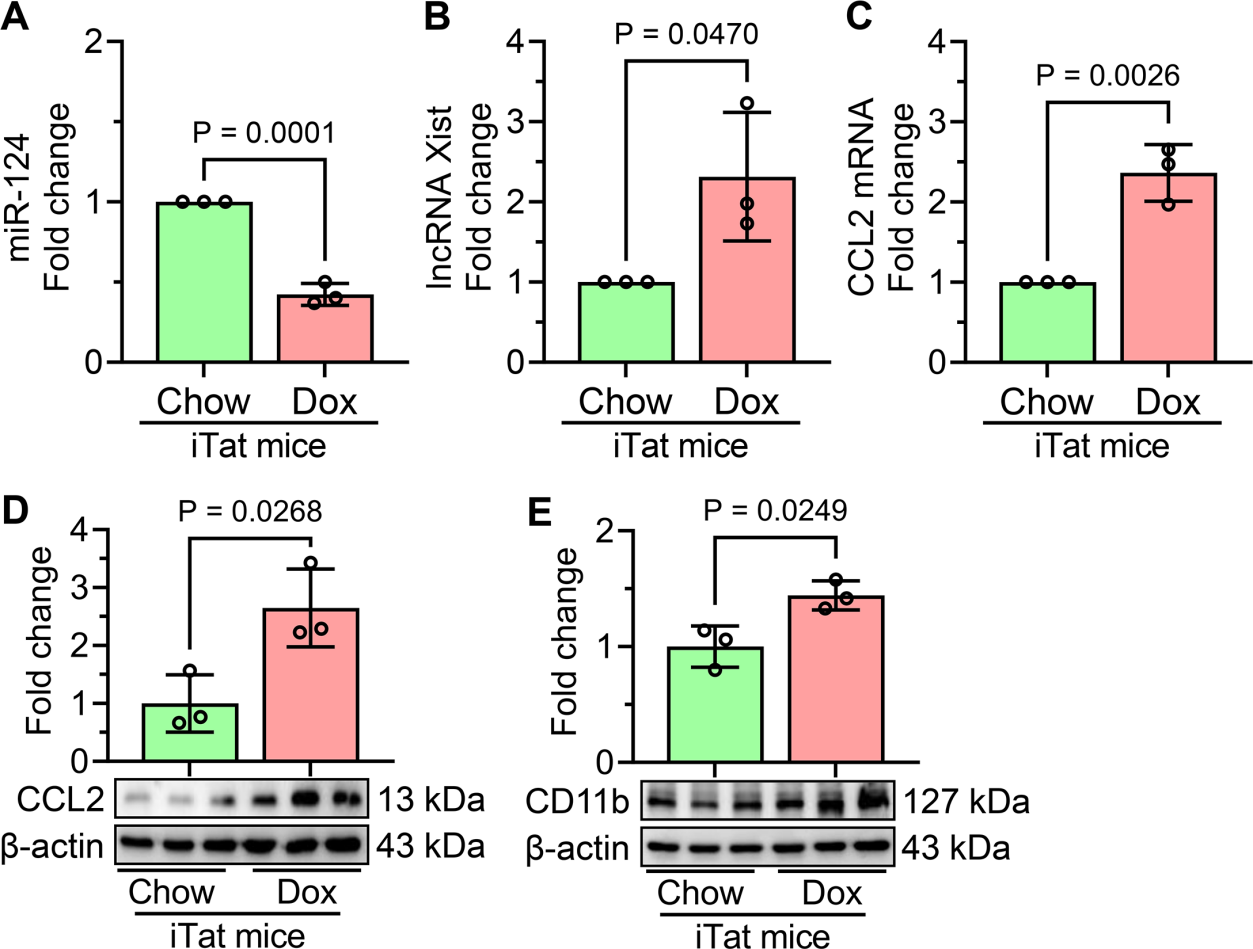
Validation of HIV Tat-mediated molecular changes in the frontal cortices of Dox-fed iTat mice. **(A)** qPCR analysis shows the expression of miR-124 in the frontal cortices of Dox-fed iTat and control mice. **(B)** qPCR analysis reveals the expression of lncRNA Xist in the frontal cortices of Dox-fed iTat and control mice. qPCR **(C)** and western blot **(D)** analyses demonstrate the increased mRNA and protein expression of CCL2 in the frontal cortices of Dox-fed iTat and control mice. **(E)** Representative western blot analysis shows the expression of microglial activation marker CD11b in the frontal cortices of Dox-fed iTat and control mice. β-actin was used as the internal control for all experiments. Data are presented as mean ± SEM from three independent experiments. Statistical significance was determined using an unpaired Student’s t-test.

To confirm the *in vitro* findings of HIV Tat-mediated microglial activation involving lncRNA Xist, miR-124, and CCL2, we extended our study to an *in vivo* model using Dox-fed iTat mice. Our *in vivo* results demonstrated that Dox-fed iTat mice exhibited a significant reduction in miR-124 expression (**Figure 6A**) in the frontal cortices, accompanied by increased levels of lncRNA Xist (**Figure 6B**). Additionally, CCL2 expression was significantly upregulated at both the mRNA and protein levels (**Figures 6C, D**). Notably, a marked increase in the microglial activation marker CD11b was also observed (**Figure 6E**) in the frontal cortices of Dox-fed iTat mice. These changes were not evident in control mice, thereby confirming the specific effects of HIV Tat induction in this model. These *in vivo* findings validate our *in vitro* results, further supporting the involvement of lncRNA Xist, miR-124, and CCL2 in HIV Tat-induced microglial activation and neuroinflammation.

## 4 Discussion

**Figure 7:**
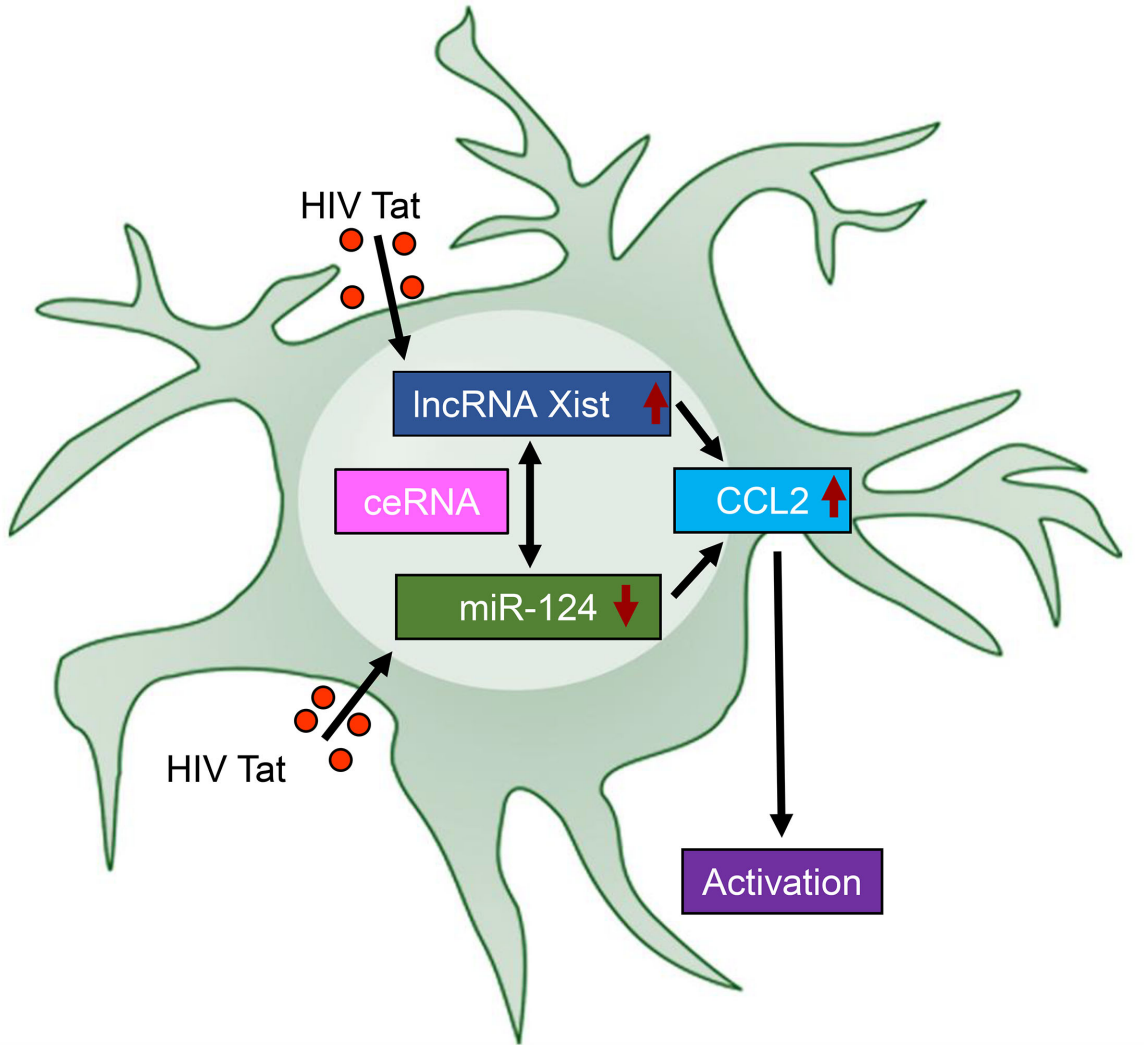
Schematic representation of the lncRNA Xist/miR-124/CCL2 axis in HIV Tat-induced microglial activation and neuroinflammation. HIV Tat exposure downregulates the expression of miR-124 while upregulating the expression of lncRNA Xist. This dysregulation acts as a competing endogenous RNA (ceRNA) to sequester miR-124, further amplifying CCL2 expression, thereby leading to microglial activation – a central feature of NeuroHIV pathogenesis – and driving neuroinflammatory responses. This schematic highlights the critical interplay among lncRNA Xist, miR-124, and CCL2 in HIV Tat-mediated neuroinflammation, offering insights into potential therapeutic targets for NeuroHIV.

HIV-associated neurocognitive disorders (HAND) remain a critical clinical challenge despite the widespread use of cART. HAND is characterized by cognitive decline, ranging from mild impairment to severe dementia, with neuroinflammation emerging as a cornerstone underlying the disease pathogenesis. Among various HIV viral proteins, Tat has been recognized as one of the key contributors to neuroinflammatory processes, based on its ability to disrupt host cellular pathways and amplify immune responses. Elevated levels of CCL2, a chemokine implicated in immune cell recruitment and neuroinflammation, have been linked to microglial activation, synaptic damage, and cognitive decline in HAND (36–38). The mechanisms underlying HIV Tat-induced CCL2 upregulation and microglial activation, however, remain incompletely understood. This study elucidated a novel regulatory mechanism involving lncRNA Xist, miR-124, and CCL2 axis in HIV Tat-induced microglial activation and neuroinflammation. Our findings demonstrated that HIV Tat significantly downregulated miR-124, a microRNA critical for maintaining microglial quiescence, while concurrently upregulating lncRNA Xist and CCL2. These molecular changes collectively drive microglial activation, a hallmark of NeuroHIV pathogenesis (**Figure 7**).

In NeuroHIV, several miRNAs, such as miR-155, miR-146a, miR-32, and miR-505, miR 138, and also miR-9 are often upregulated, promoting chronic inflammation (33, 39–43). For instance, miR-155 is linked to proinflammatory responses by enhancing the production of cytokines such as TNF-α and IL-6, which exacerbate neurotoxic effects (44). In contrast, miR-124 is downregulated in NeuroHIV and other neurodegenerative conditions (8), leading, in turn, to excessive microglial activation and neuroinflammation. miR-124 is highly expressed in the CNS and plays a pivotal role in maintaining the anti-inflammatory phenotype of microglia by targeting proinflammatory signaling pathways such as NF-κB, STAT3, and TLR4 (18, 19, 45). Its dysregulation in NeuroHIV and other neurodegenerative diseases has been associated with excessive activation of microglia and increased production of inflammatory mediators, including CCL2 (46–48). In our study, miR-124 directly targeted the 3′-UTR of CCL2, as demonstrated by bioinformatics analysis, Argonaute immunoprecipitation, and functional assays. HIV Tat-induced downregulation of miR-124 led to de-repression of CCL2, resulting in its upregulation at both transcript and protein levels. This aligns with prior research showing that restoring miR-124 levels attenuated inflammatory responses and suppressed CCL2 expression in various disease contexts (49, 50). The epigenetic regulation of miR-124 expression adds another layer of complexity. In this regard, our previous studies suggest that HIV Tat promoted DNA methylation of miR-124 gene loci via modulating DNA methyltransferase enzymes, thereby contributing to its downregulation (19). Additional mechanisms, such as histone deacetylation, have also been implicated and shown to suppress miR-124 expression, with histone deacetylase inhibitors effectively restoring its levels (49). These findings thus underscore the potential of targeting epigenetic pathways to rescue miR-124 expression and mitigate neuroinflammation.

LncRNAs have emerged as crucial regulators of gene expression and inflammation, often acting as ceRNAs that sequester miRNAs and modulate their activity (51). Among these, lncRNA Xist has been implicated in various inflammatory and neurodegenerative conditions, including cerebral infarction and Alzheimer’s disease (27, 28). Our current study identified lncRNA Xist as a key mediator of HIV Tat-induced microglial activation involving the ceRNAs mechanism(s). HIV Tat exposure significantly upregulated lncRNA Xist expression in mouse primary microglia in a dose- and time-dependent manner. By acting as a ceRNA, lncRNA Xist sequestered miR-124, thereby reducing its availability to target CCL2. This sponging effect was validated through luciferase reporter assays and siRNA-mediated silencing of lncRNA Xist, which restored miR-124 levels, suppressed CCL2 expression, and attenuated microglial activation. The lncRNA Xist-miR-124-CCL2 axis thus represents a critical regulatory pathway in NeuroHIV-related neuroinflammation.

Recent studies have also identified over 40 lncRNA-miR-124-mRNA interactomes that utilize this competitive binding mechanism, particularly in the context of various epithelial cancers (52). Among these lncRNAs, MALAT1, NEAT1, HOXA11-AS, and lncRNA Xist are the most prominent and frequently studied within these regulatory networks (52). Notably, the lncRNA Xist, best known for its role in X-chromosome inactivation, has recently gained attention as a regulator of neuroinflammation and immune responses, positioning it as a potential contributor to neuroinflammatory disorders. LncRNA Xist influences the expression of proinflammatory cytokines and key signaling pathways, including the NFκB (53), which are critical for microglial activation during HIV infection. Interestingly, the inhibition of lncRNA Xist has also been shown to promote M2 polarization of microglia, which paradoxically exacerbates spinal cord injury. This effect is mediated through the regulation of the miR-124-3p-IRF1 axis, thus underscoring the complex interplay between the lncRNA Xist-miR-124 axis and immune modulation in disease progression (29). Interestingly, similar ceRNA mechanisms involving lncRNA Xist have been reported in cancer and other inflammatory conditions. For example, lncRNA Xist modulates neuroinflammatory responses by interacting with miR-96-5p to regulate NF-κB signaling in cerebral infarction (27). Its role in immune modulation further underscores the potential therapeutic relevance of targeting lncRNA Xist in NeuroHIV. The *in vivo* relevance of the lncRNA Xist-miR-124-CCL2 axis was confirmed using dox-fed iTat mice. In these mice, HIV Tat expression was associated with downregulated expression of miR-124 levels, upregulated expression of lncRNA Xist and CCL2, that was accompanied with upregulation of microglial activation markers such as CD11b, in the frontal cortices of mice. These findings mirror our *in vitro* findings, thus validating the physiological significance of this regulatory axis in the pathogenesis of NeuroHIV. Although our *in vitro* data specifically focus on microglial cells, the *in vivo* validation was conducted using total brain cortical tissue, which includes other CNS cell types in addition to microglia. Therefore, these results should be interpreted in that context, and future studies using cell-type-specific sorting or labeling are warranted. It is also noted that while CD11b is a commonly used marker of microglial activation, it is not entirely specific to microglia, as it can also be expressed by other myeloid cells. In this study, its use is supported by prior validation of microglial purity (>95%) in our cultures using Iba1 immunostaining.

Targeting the lncRNA Xist-miR-124-CCL2 axis offers promising adjunctive therapeutic potential for mitigating neuroinflammation in NeuroHIV. Approaches such as antisense oligonucleotides or siRNA to silence lncRNA Xist could likely restore miR-124 levels and suppress CCL2 expression, thereby reducing microglial activation and its downstream effects. Similarly, delivering miR-124 mimics or using CRISPR-Cas9 technology to inhibit lncRNA Xist expression could counteract the proinflammatory effects of this pathway. Moreover, combining these strategies with cART could address both viral persistence and neuroinflammatory pathways, providing a comprehensive approach to NeuroHIV management. Given the critical role of epigenetic regulation in miR-124 expression, exploring the use of DNA methyltransferase inhibitors or histone deacetylase inhibitors could further enhance the therapeutic outcomes.

Despite the insights gained, this study has certain limitations that warrants further consideration. First, while we demonstrated the involvement of the lncRNA Xist-miR-124-CCL2 axis in microglial activation and neuroinflammation, the exact upstream mechanisms driving HIV Tat-induced lncRNA Xist upregulation still remain unclear. Further studies are needed to explore whether epigenetic modifications, transcriptional regulators, or RNA-binding proteins contribute to lncRNA Xist dysregulation. Second, although the findings were validated in the frontal cortices of Dox-fed iTat mice, the use of a single viral protein *in vivo* model limits the generalizability of our conclusions. Additional studies using alternative animal models of NeuroHIV, such as simian immunodeficiency virus (SIV)-infected macaques, would provide broader validation. Finally, this study focused on the molecular interplay among lncRNA Xist, miR-124 and CCL2, but the potential involvement of other regulatory pathways or interacting molecules in HIV Tat-mediated neuroinflammation remains unexplored. Addressing these limitations in future research could provide a more comprehensive understanding of NeuroHIV pathogenesis. For example, how NLRP3 fits in this axis is an area that needs investigation. Future research should focus on elucidating the upstream regulators of lncRNA Xist in HIV infection, including the potential roles of epigenetic modifications and transcriptional regulators. In parallel, investigating other lncRNA–miRNA regulatory networks involved in NeuroHIV could reveal broader mechanisms underlying neuroinflammation. Preclinical studies evaluating the efficacy of therapeutics targeting the lncRNA Xist–miR-124–CCL2 axis in diverse animal models of NeuroHIV are essential for advancing these findings toward clinical application. Finally, future studies will aim to validate this axis in post-mortem brain tissues from cART-treated HIV-positive individuals to establish its clinical relevance. In summary, this study identifies the lncRNA Xist-miR-124-CCL2 axis as a novel regulatory pathway in HIV Tat-induced neuroinflammation. By elucidating the molecular mechanisms driving microglial activation, these findings provide new insights into NeuroHIV pathogenesis and highlight potential therapeutic targets to mitigate neuroinflammation and cognitive decline in PLWH.

## Data Availability

The raw data supporting the conclusions of this article will be made available by the authors, without undue reservation.
